# Pharmacometabolomics for predicting variable busulfan exposure in paediatric haematopoietic stem cell transplantation patients

**DOI:** 10.1038/s41598-017-01861-7

**Published:** 2017-05-10

**Authors:** Bora Kim, Ji Won Lee, Kyung Taek Hong, Kyung-Sang Yu, In-Jin Jang, Kyung Duk Park, Hee Young Shin, Hyo Seop Ahn, Joo-Youn Cho, Hyoung Jin Kang

**Affiliations:** 1 Department of Clinical Pharmacology and Therapeutics, Seoul National University College of Medicine and Hospital, Seoul, Korea; 2 Department of Pediatrics, Cancer Research Institute, Seoul National University College of Medicine and Hospital, Seoul, Korea; 3 Department of Pediatrics, Samsung Medical Center, Sungkyunkwan University School of Medicine, Seoul, Korea

## Abstract

Owing to its narrow therapeutic range and high pharmacokinetic variability, optimal dosing for busulfan is important to minimise overexposure-related systemic toxicity and underexposure-related graft failure. Using global metabolomics, we investigated biomarkers for predicting busulfan exposure. We analysed urine samples obtained before busulfan administration from 59 paediatric patients divided into 3 groups classified by area under the busulfan concentration-time curve (AUC), i.e., low-, medium-, and high-AUC groups. In the high-AUC group, deferoxamine metabolites were detected. Phenylacetylglutamine and two acylcarnitines were significantly lower in the high-AUC group than in the low-AUC group. Deferoxamine, an iron-chelating agent that lowers serum ferritin levels, was detected in the high-AUC group, indicating that those patients had high ferritin levels. Therefore, in a retrospective study of 130 paediatric patients, we confirmed our hypothesis that busulfan clearance (dose/AUC) and serum ferritin level has a negative correlation (r = −0.205, P = 0.019). Ferritin, acylcarnitine, and phenylacetylglutamine are associated with liver damage, including free radical formation, deregulation of hepatic mitochondrial β-oxidation, and hyperammonaemia. Our findings reveal potential biomarkers predictive of busulfan exposure and suggest that liver function may affect busulfan exposure.

## Introduction

Busulfan is one of the most frequently used chemotherapeutic agents as a conditioning regimen before hematopoietic stem cell transplantation (HSCT) for various malignant and non-malignant diseases. It has a narrow therapeutic range with a risk of toxicities after high exposures, such as veno-occlusive disease^1^. Higher busulfan exposures are also associated with lower relapse rates among patients with previously untreated chronic myeloid leukaemia^2^ as well as lower rates of graft failure^3^. Busulfan pharmacokinetics (PK) are known to be variable even after the use of intravenous (IV) busulfan, especially in children^4^. Personalized dosing of busulfan using therapeutic drug monitoring (TDM) has been recommended because of its narrow therapeutic range and variable PKs; evidence-based guidelines for personalizing busulfan-based conditioning have been developed by the American Society for Blood and Marrow Transplantation^5^.

Previously, we performed a Phase I clinical study to determine the optimal once-daily busulfan dose using PK modelling. That study evaluated PK characteristics of a once-daily busulfan dose for four consecutive days. The daily targeted area under the curve (AUC) was set at 18,000–19,000 μg∙h/L/day to reduce graft failure and improve HSCT outcomes^6^. The clinical application of busulfan TDM is still challenging. Dose adjustment after busulfan TDM during conditioning chemotherapy is labour intensive because it requires frequent sampling and appropriate institutional facilities. However, some patients have been considerably under- or over-dosed by the initial (i.e., before TDM results are available) dose of busulfan. As a surrogate method, the initial dose of busulfan is calculated according to body weight (mg/kg) or body surface area (mg/m^2^). However, body surface area cannot predict the large inter-individual variations in busulfan PKs, which explains the risk of busulfan over- or under-dosing on the first day.

To reduce the variability in busulfan exposure, several studies were performed to personalise busulfan dosing. Busulfan is metabolised in the liver by glutathione S transferase (GST) enzymes, primarily GSTA1, followed by GSTM1, GSTP1, and GSTT1^7–9^. However, there are conflicting data regarding the association between busulfan PKs and GST polymorphisms. Some studies have demonstrated positive associations between GSTs and busulfan PKs^7, 10, 11^, whereas others have not^12–14^. Presently, pharmacogenomics-based busulfan dosing is not recommended for routine clinical practice^5^. Population PK modelling of intravenous busulfan administration has indicated that age and body size (body weight or body surface area) are associated with clearance in children^15^. Currently, we cannot elucidate all sources of variation in drug response phenotypes with genetics alone. Additional factors, such as environment, age, ethnicity, and the use of other medications, strongly contribute to variations in drug response. Concomitant medications administered during conditioning chemotherapy have been shown to interact with busulfan. Common concomitant medications include antibiotics (e.g. metronidazole), antifungal agents (e.g. itraconazole and fluconazole), seizure prophylactics (e.g. phenytoin), analgesics (e.g. ketobemidone), and antipyretics (e.g. acetaminophen). It has been reported that these medications affect busulfan PKs and outcomes by increasing busulfan exposure^16–20^. The exact cause of such interactions remains unknown; however, it is often attributed to either inhibition or induction of cytochrome P450s (CYPs), depletion of glutathione (GSH), or altered function of drug transporters.

Pharmacometabolomics is an emerging “omics” field that is focused on the use of individual metabolic signatures to define mechanisms of action and variations in response to treatment, supporting personalized drug therapy^21^. The metabolome, which represents both the downstream output of the genome and upstream input from the environment, can provide comprehensive insights into the form of endogenous (gene-derived) metabolites and exogenous (environment-derived) metabolites that can explain individual phenotypic variations. With a focus on precision medicine, pharmacometabolomics uses individual metabolic signatures to predict or evaluate variations in drug responses (efficacy and toxicity) and explain the underlying mechanisms behind variable patient responses^22–26^, verifying it as a useful platform for individualized drug therapy.

This study identifies potential biomarkers for predicting individual variations in busulfan PKs using untargeted metabolomics and suggests a mechanism for busulfan PK variability.

## Results

### Inter-individual variability

**Figure 1:**
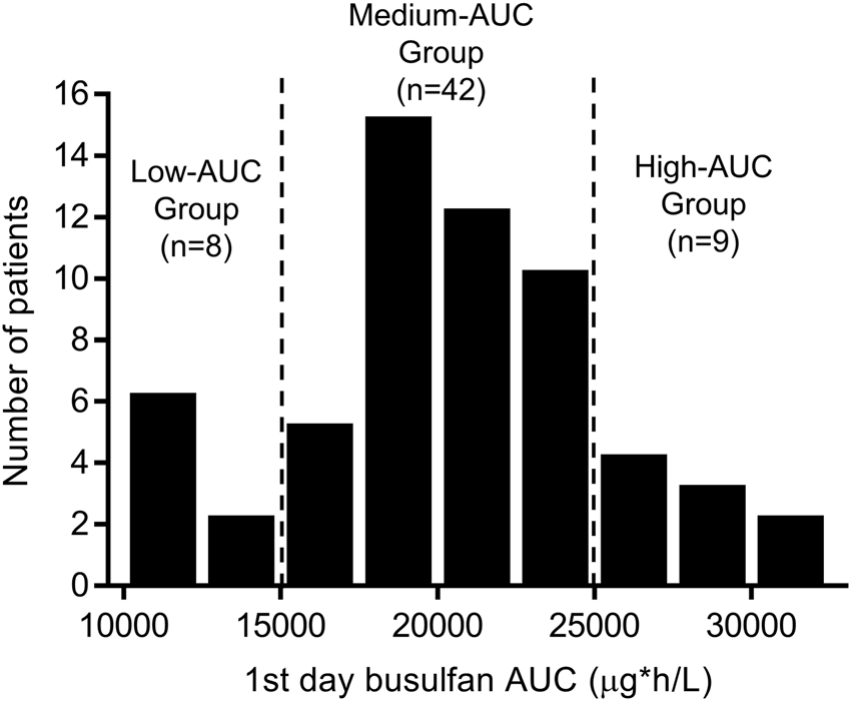
Frequency distribution of the busulfan area under the curve (AUC) on the first day in 59 paediatric patients. The AUC ranged from 11,668 to 31,197 μg·h/L (median 20,037 μg·h/L, % covariate =23.5%) after infusing 120 mg/m^2^ busulfan on the first day.

We recruited 59 paediatric patients (see Table 1 for demographics) for metabolomic profiling in which the AUC ranged from 11,668 to 31,197 μg·h/L (median 20,037 μg·h/L, % covariate (CV) = 23.5%) after infusing 120 mg/m^2^ busulfan on the first day (Fig. 1). To find metabolic markers predicting variable busulfan exposure, the patients were divided into 3 pharmacokinetic response groups according to their busulfan AUCs on the first day of administration as follows: the low-AUC group (n = 8) had an AUC lower than 15,000 μg·h/L, the medium-AUC group (n = 42) had an AUC between 15,000–25,000 μg·h/L, and the high-AUC group (n = 9) had an AUC greater than 25,000 μg·h/L. The grouping criteria (15,000 and 25,000 μg·h/L) were determined according to the lowest and highest 15% of busulfan AUCs for of each group on the first day.

**Table 1:** Demographics.

Characteristics	Pharmacometabolomics study (n = 59)	Retrospective study (n = 130)
**Median age, yr (range)**	12.3 (1.3–22.2)	9.5 (0.6–22.2)
**Gender, No. (%)**		
Male	35 (59.3)	69 (53.1)
Female	24 (40.7)	61 (46.9)
**Diagnosis, No. (%)**		
Acute leukaemia	40 (67.8)	92 (70.8)
Acute lymphoblastic leukaemia	20 (33.9)	41 (31.5)
Acute myeloid leukaemia	17 (28.8)	44 (33.8)
Other	3 (5.1)	7 (5.4)
Lymphoma	2 (3.4)	3 (2.3)
Haematological disease	4 (6.8)	11 (8.5)
Myelodysplastic syndrome	1 (1.7)	3 (2.3)
Haemophagocytic lymphohistiocytosis	1 (1.7)	3 (2.3)
Severe aplastic anaemia	1 (1.7)	1 (0.7)
Paroxysmal nocturnal haemoglobinuria	1 (1.7)	1 (0.7)
Solid tumour	7 (11.9)	11 (8.5)
Immunodeficiency	4 (6.8)	9 (6.9)
Other diseases*	2 (3.4)	4 (3.1)
**Conditioning regimen, No. (%)**		
Busulfan fludarabine etoposide	19 (32.2)	58 (44.6)
Busulfan fludarabine	18 (30.5)	52 (40.0)
Busulfan fludarabine melphalan	6 (10.2)	6 (4.6)
Busulfan melphalan	6 (10.2)	10 (7.7)
Other	0 (0.0)	4 (3.1)

### Metabolic profiling of predose urine samples

**Figure 2:**
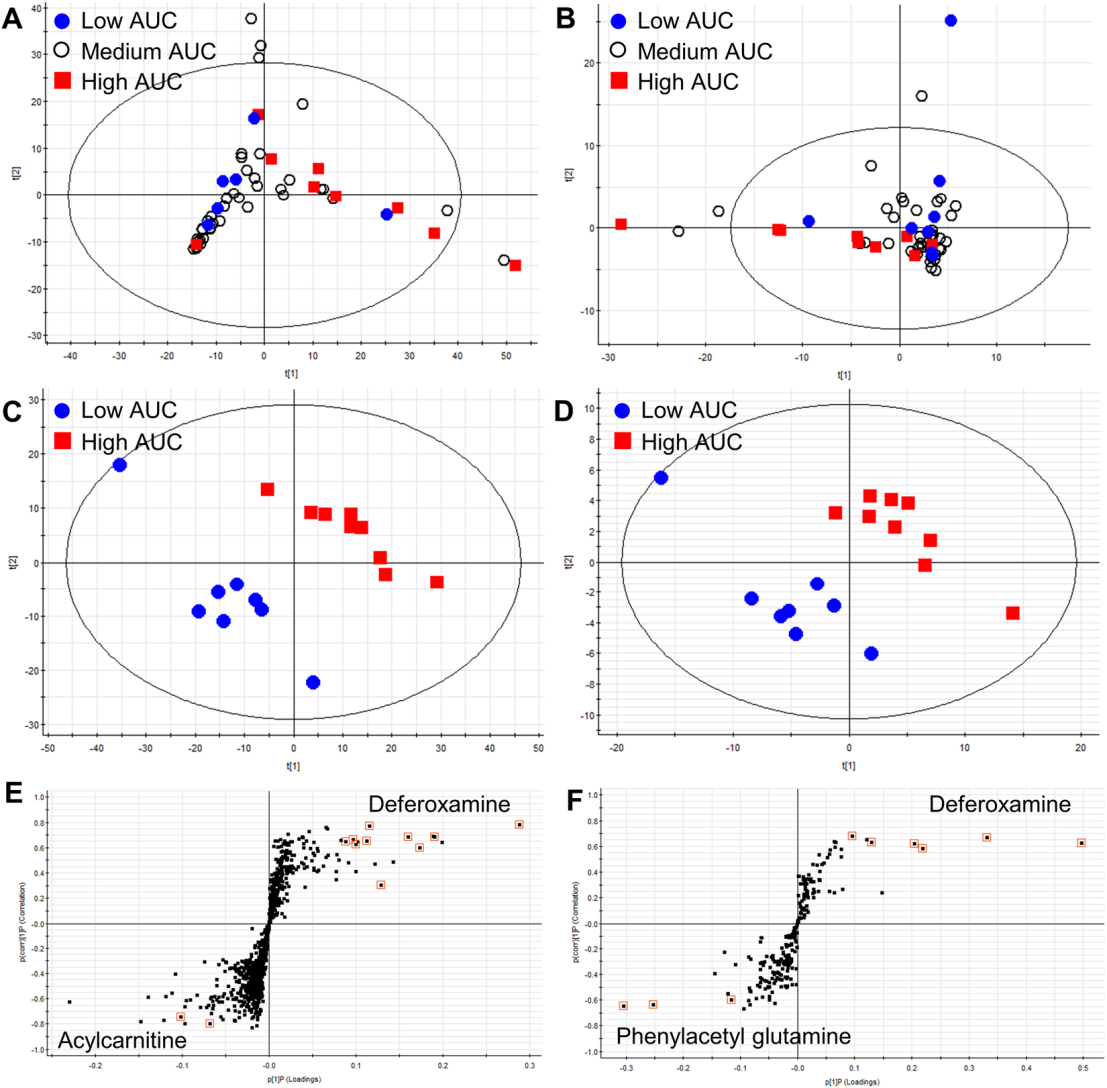
Sixteen deferoxamine-derived metabolites, two acylcarnitines, and phenylacetylglutamine were identified by pharmacometabolomics and significantly associated with high exposure to busulfan. Two dimensional unsupervised principle component analyses in (**A**) positive and (**B**) negative ion mode were conducted. Supervised partial least-squares to latent structure discriminant analysis (PLS-DA) of (**C**) positive and (**D**) negative ion mode, and the orthogonal PLS-DA following S-plot of (**E**) positive and (**F**) negative ion mode illustrated the putative metabolites responsible for discrimination of the low-AUC group from the high-AUC group.

From untargeted metabolomics, a total of 2,477 metabolic features were detected in the ESI^+^ and ESI^−^ mode. Principal component analysis (PCA), an unsupervised multivariate statistical approach, showed no clear differences between the low-, medium-, and high-AUC groups; however, the high-AUC group had a tendency to cluster, indicating similar metabolic features between the samples (Fig. 2A,B). Partial least-squares to latent structure discriminant analysis (PLS-DA) score plots were obtained to show a reasonable separation between the low-AUC and high-AUC groups. PLS-DA plots (Fig. 2C,D) show that the urinary profiles could be separated according to the busulfan AUC group (ESI^+^ mode: goodness of fit (R^2^) = 0.998 and predictability (Q^2^) = 0.821; ESI^−^ mode: R^2^ = 0.548 and Q^2^ = 0.304). R^2^ and Q^2^ were relatively lower in negative ion mode than positive ion mode. To discern which metabolites were most responsible for the separation between the low- and high-AUC groups, a supervised orthogonal PLS-DA analysis was performed; the findings are expressed in a loading S-plot (Fig. 2E,F). Potential marker metabolites for predicting busulfan AUCs were selected using S-plots and variable importance in the projection (VIP) statistics. In the S-plot, 16 metabolites (right upper quadrant in both ESI^+^ and ESI^−^ mode) were identified as deferoxamine-derived metabolites that indicated a relatively higher abundance in the high-AUC group than that of the low-AUC group (Fig. 2E,F). These metabolites showed the same tandem mass spectrometry (MS/MS) fragmentation patterns (m/z 84.0818, 102.0924, 144.1054, 201.1244, and 243.1348), which are also the fragmentation patterns of deferoxamine. Supplementary Fig. S1A shows the structure of deferoxamine and its MS/MS fragmentation pattern. The MS/MS spectrum of urinary deferoxamine was confirmed by comparing it with the authentic compound (Supplementary Fig. S1B). Two acylcarnitines (carnitine C9:1 and carnitine C12:1-OH) and phenylacetylglutamine were down-regulated in the high-AUC group (Fig. 2E,F). The MS/MS spectra of the identified metabolites are indicated in Supplementary Figs S2–S4. In the absence of reference standards for unsaturated acylcarnitines, two acylcarnitines were identified by elemental composition (MassLynx) and comparisons of MS fragmentation patterns with related saturated acylcarnitines (Supplementary Figs S2 and S3).

### Statistical analysis of metabolic markers

**Figure 3:**
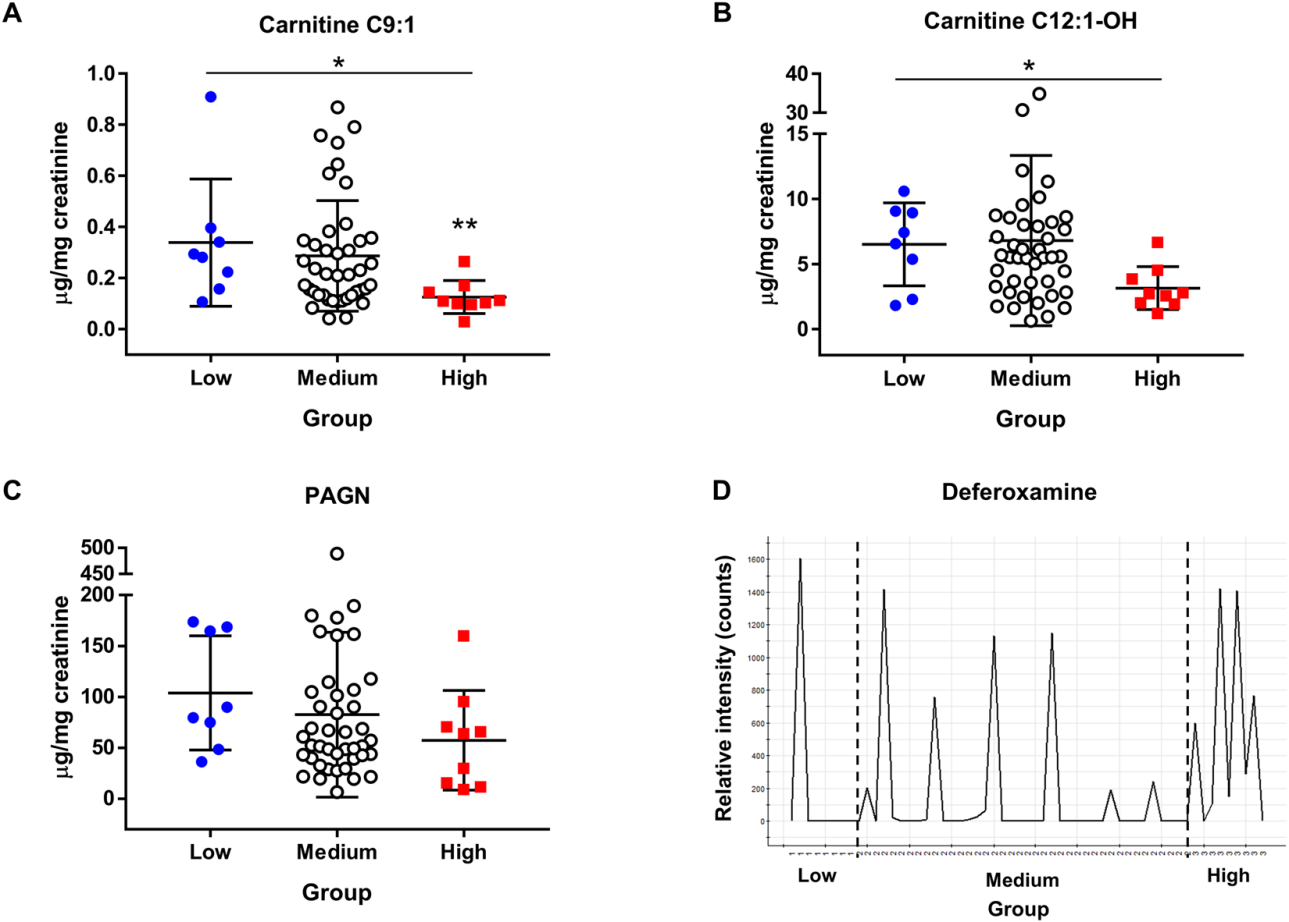
Concentrations of endogenous metabolic markers in the low-, medium, and high-AUC group. (**A**) C9:1 carnitine, (**B**) C12:1-OH carnitine, and (**C**) phenylacetylglutamine. (**D**) Relative intensity of deferoxamine in the low-, medium, and high-AUC group. *Jonckheere-Terpstra trend test (*P* < 0.05) and **Kruskal-Wallis test followed by Mann-Whitney tests (significance was adjusted by Bonferroni’s method, *P* < 0.017).

Among the identified metabolites, we semi-quantified three endogenous biomarkers (i.e. carnitine C9:1, carnitine C12:1-OH, and phenylacetylglutamine). The three endogenous metabolites were further investigated using univariate statistics (Kruskal Wallis test followed by Bonferroni-adjusted Mann-Whitney test and Jonckheere-Terpstra trend test). The Jonckheere-Terpstra’s trend test demonstrated a significantly decrease in the concentration of carnitine C9:1 (*P* = 0.004) and carnitine C12:1-OH (*P* = 0.019) from the low-, medium-, and high-AUC groups (Fig. 3A,B). In the high-AUC group, the urinary concentration of carnitine C9:1 was significantly lower than that of the low-AUC group (Kruskal Wallis test: *P* = 0.004). Further, carnitine C12:1-OH (Kruskal Wallis test: P = 0.019) and phenylacetylglutamine were lower in the high-AUC group, albeit not significantly, than those of the low-AUC group (Kruskal Wallis test: *P* = 0.065, Fig. 3A–C). Deferoxamine was administered within 30 days of busulfan treatment. Therefore, the concentration of deferoxamine did not correlate with busulfan exposure. However, the number of patients who received deferoxamine was significantly different between the low- and high-AUC groups. Seven patients in the high-AUC group (total group n = 9) and one patient in the low-AUC group (total group n = 8) received deferoxamine, and its metabolite was detected in the urine (Fig. 3D). The identified biomarkers are listed in Table 2.

**Table 2:** Identified metabolites that were significantly different between low- and high-area under the curve (AUC) groups.

Metabolites	Ion mode	Mass (*m/z*)	RT (min)	P (Loadings)	P (correlation)	VIP	Concentration (μg/mg creatinine, mean ± SD)			Low vs High (*p* value)^†^	Trend test (*p* value)^‡^
							Low	Medium	High		
Carnitine C9:1	ESI+	300.2180*	10.45	−0.07	−0.80	2.36	0.34 ± 0.25	0.29 ± 0.22	0.13 ± 0.06	0.006	0.004
Carnitine C12:1-OH	ESI+	358.2590*	12.06	−0.10	−0.74	3.59	6.51 ± 3.18	6.80 ± 6.54	3.15 ± 1.65	0.059	0.019
PAGN	ESI−	263.1032*	4.60	−0.30	−0.65	4.76	103.96 ± 56.12	82.67 ± 80.90	57.45 ± 48.93	0.059	0.065
	ESI−	145.0613	4.60	−0.25	−0.64	4.10					
	ESI−	549.1961	4.60	−0.12	−0.60	1.87					
Deferoxamine-derived metabolites	ESI +	629.2350	6.50	0.29	0.78	9.67					
	ESI−	574.3083	9.87	0.50	0.62	7.73					
	ESI+	614.2720	4.20	0.19	0.69	7.08					
	ESI+	598.3060	9.87	0.17	0.60	6.41					
	ESI+	401.2390	7.86	0.16	0.69	5.56					
	ESI+	561.3600	7.90	0.13	0.30	4.95					
	ESI−	399.2237	7.86	0.33	0.67	4.21					
	ESI+	601.2040	5.33	0.11	0.65	4.17					
	ESI+	651.2180	6.49	0.12	0.77	3.87					
	ESI+	483.2430	8.50	0.10	0.63	3.83					
	ESI+	522.2900	7.74	0.10	0.66	3.40					
	ESI−	546.2764	8.98	0.22	0.58	3.34					
	ESI−	459.2447	8.48	0.20	0.62	3.20					
	ESI+	201.1240	7.87	0.09	0.65	3.11					
	ESI−	596.2913	9.86	0.13	0.63	1.96					
	ESI−	498.2924	7.73	0.10	0.68	1.32					

*Adducts of [M + H] + or [M − H]- for quantification, ^†^Kruskal Wallis test followed by Mann-Whitney test (Significance was adjusted by Bonferroni’s method, *P* < 0.017), ^‡^Jonckheere-Terpstra trend test (*P* < 0.05); PAGN, phenylacetylglutamine; RT, real time; VIP, variable importance in the projection.

### Ferritin levels and busulfan clearance

**Figure 4:**
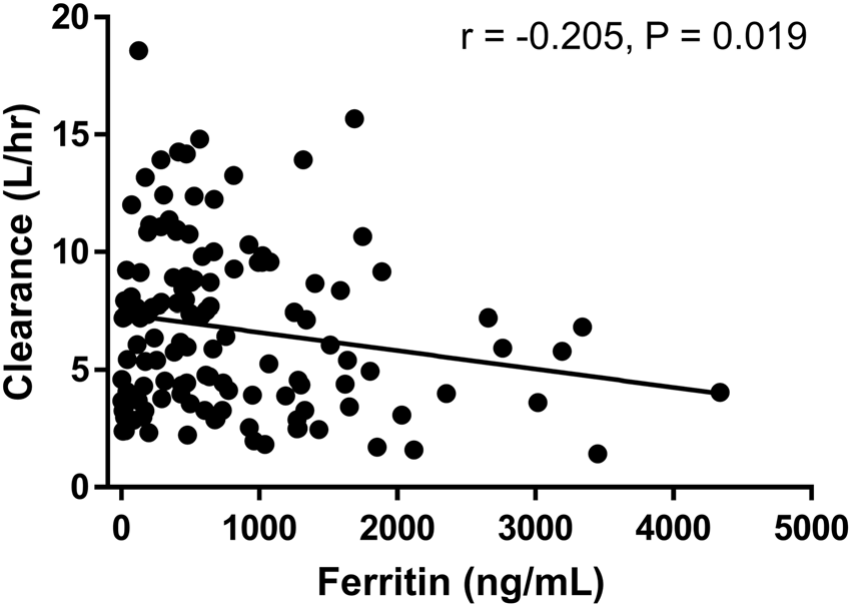
(**A**) The first day area under the curve (AUC) and (**B**) clearance showed gradual elevations or decreased along with the ferritin level when patients were divided into 4 groups as follows: ferritin <500, ≥500–1,000, ≥1,000–2,000, and ≥2,000 ng/mL.

In our institution, ferritin levels were measured monthly during the chemotherapy or supportive care that occurs before HSCT because those patients are at an increased risk of iron overload attributable to previous transfusions. Deferoxamine was administered when serum-ferritin levels were greater than 500 ng/mL and used until the infusion day in patients whose ferritin levels continued to be above 500 ng/mL. Deferoxamine metabolites were detected in the high-AUC group, likely attributable to high ferritin levels. To confirm this, we analysed the association among ferritin levels and first day busulfan clearance in 130 paediatric patients. Patient demographics are summarized in Table 1. The first day busulfan clearance (L/hr) showed a negative correlation with the ferritin level (r = −0.205, *P* = 0.019) in the correlation analysis result (Fig. 4).

## Discussion

This study identifies urinary biomarkers that predict busulfan exposure in HSCT paediatric patients using pharmacometabolomics. Recently, pharmacometabolomics-based endogenous plasma markers associated with IV busulfan clearance were evaluated in adult HSCT recipients, and a seven-ion linear model was developed to predict IV busulfan clearance^27^. Here, we report the effectiveness of using pharmacometabolomics to establish markers that reflect both intrinsic and extrinsic factors, such as the use of other drugs. Patients undergoing HSCT have complex medical conditions such as disease subtype, complications, and use of other medications. Thus, concomitant medications or medical indications can affect PKs and outcomes of target drugs.

We demonstrated that deferoxamine and its metabolites correlate with the busulfan AUC, which provides insight into the association between serum ferritin levels and busulfan PKs for deferoxamine-treated patients with serum ferritin levels (≥500 ng/mL). In this study, we found a negative correlation between busulfan clearance and ferritin levels before HSCT, which indicate that busulfan metabolism is decreased in patients with high ferritin levels. Clinically, serum ferritin levels >1,000 ng/mL are associated with iron overload and liver disease^28^. Moreover, it has been shown clinically that ferritin levels affect HSCT patients, as hyperbilirubinaemia and treatment-related mortality are significantly higher in HSCT patients with ferritin levels >1,000 ng/mL^29^. When patients were divided into two groups according to ferritin levels (ferritin ≥1,000 or <1,000 ng/mL), the first day AUC (μg·h/L)/dose (mg/m^2^) ratio was significantly higher, while the clearance (L/h) was significantly lower in patients whose ferritin levels were ≥1,000 ng/mL than those of the remaining patients (AUC/dose values were 192.5 ± 57.0 vs 165.3 ± 38.4, *P* = 0.008; clearance values were 5.6 ± 3.4 L/h vs 7.2 ± 3.6 L/h, *P* = 0.017). In patients older than 1 year who received 120 mg/m^2^ of busulfan on the first day, the optimal busulfan dose to meet the target AUC (18,750 μg·h/L/day) was 119.7 ± 30.1 mg/m^2^ in patients with ferritin <1,000 ng/mL and 106.1 ± 29.3 mg/m^2^ in patients with ferritin ≥1,000 ng/mL (*P* = 0.021). These findings suggest that a general once daily dose of busulfan (120 mg/m^2^ for patients ≥1 year) is optimal for patients with ferritin levels <1,000 ng/mL, but may cause busulfan overexposure in patients with ferritin levels ≥1,000 ng/mL. However, these alternative busulfan dosing schema should be validated in further dataset.

**Figure 5:**
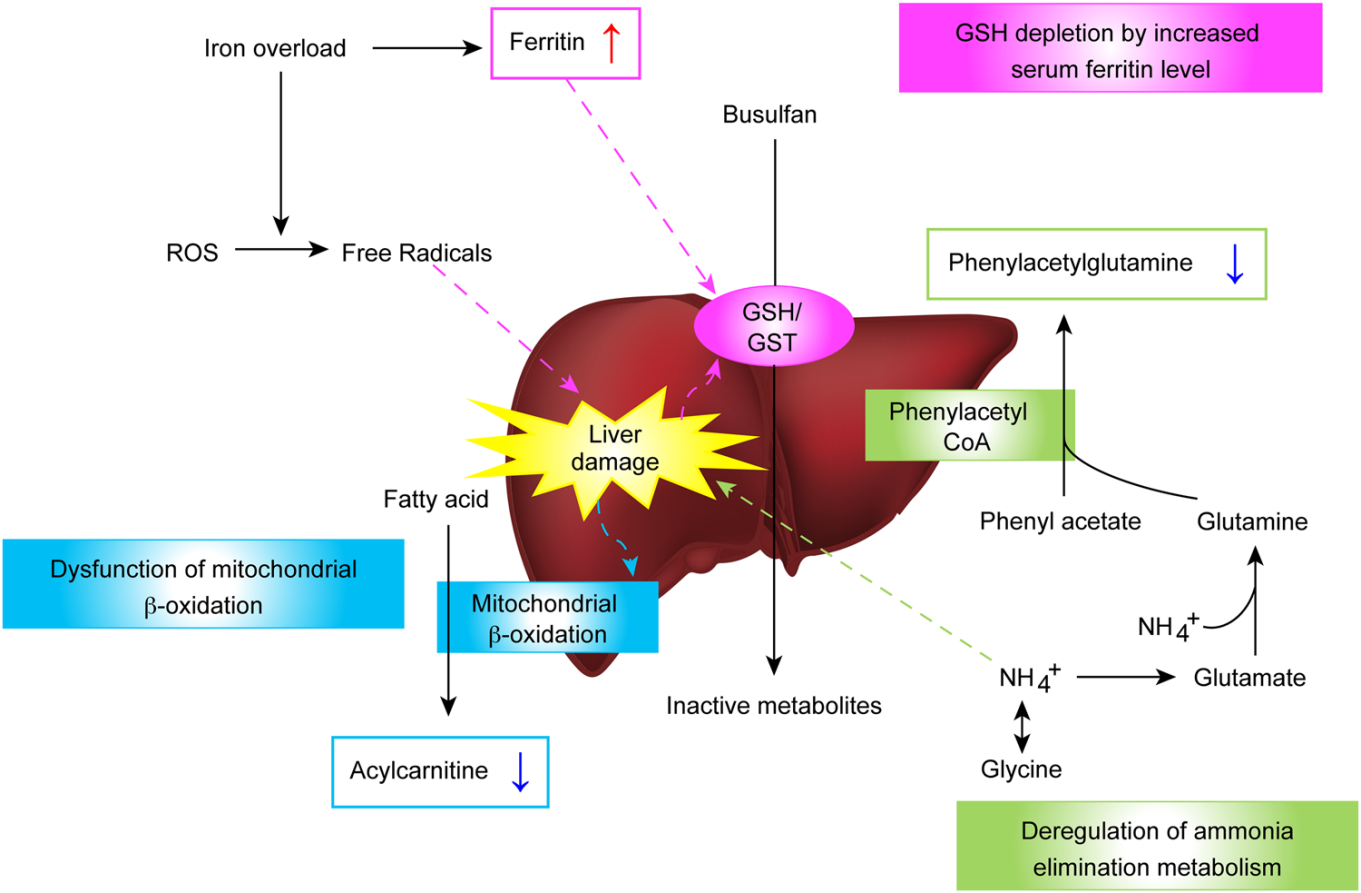
Potential mechanism for busulfan overexposure, including liver damage related markers.

Decreased busulfan metabolism in patients with high ferritin levels can possibly be explained by the significant correlation between serum ferritin levels and liver iron storage. Elevated serum ferritin is a surrogate marker of iron overload and iron toxicity. Patients who undergo HSCT are at increased risk of iron overload attributable to previous transfusions. Once iron is absorbed, there are no physiological mechanisms for excretion of excess iron from the body. Normally, iron is excreted through sweat, shed skin cells, and the gastrointestinal tract at a rate of approximately 1 mg/day, which is similar to the rate of iron absorption^30^. In transfusion-dependent patients and in the absence of this regulating mechanism, iron overload can occur. The iron-chelating agent, deferoxamine, has been used in patients with iron overload. During conditioning chemotherapy, the amount of free iron increases because erythrocytes are not utilizing iron and it is not being released from destroyed cells^31^. This free iron catalyses the conversion of reactive oxygen species (ROS) intermediates to highly toxic free radicals, which cause tissue damage^32^. Liver damage attributable to iron overload could be a mechanism of decreased busulfan metabolism in patients with high ferritin levels because busulfan is primarily metabolized in the liver through conjugation by GST-family enzymes. Moreover, previous studies have demonstrated that increased serum ferritin levels might be responsible for activation of GSH turnover, causing a reduction in both plasma and erythrocyte GSH levels; this reduction could be related to decreased busulfan metabolism (Fig. 5)^33, 34^. However, this interpretation must be carefully applied, as the correlation coefficient between liver iron content (LIC), as estimated by magnetic resonance imaging (MRI), and serum ferritin is approximately 0.6 to 0.8, implying that ferritin is an acceptable but imperfect surrogate of true iron burden^35^. In recent studies, iron overload itself (as estimated by LIC) is not associated with an increase in mortality even though high ferritin levels are significantly associated with mortality^35, 36^. These results indicate that hyperferritinaemia rather than iron overload is the more important factor for predicting HSCT outcomes. There are many other conditions, such as infection and inflammation, that increase serum ferritin levels, and ferritin levels can vary depending on the physical condition. In addition to iron overload, these various conditions should be considered as factors that can influence busulfan PKs. Further studies are needed to clarify this issue.

Two previous population PK studies of busulfan in paediatric patients incorporated ferritin levels as a covariate. Busulfan PKs were not influenced by ferritin levels in these studies^37, 38^. However, one study examined thalassemia patients^37^ and the other examined patients with non-malignant diseases^38^. These patient populations are different from the population examined in our study in which the majority had a malignant disease and a history of previous chemotherapy. In addition, those previous studies primarily included patients with pre-existing liver damage and/or iron overload. The differing results regarding the influence of ferritin levels on busulfan PKs observed in the present study may result from differences in the baseline characteristics of enrolled patients.

Many previous studies have demonstrated adverse impacts of elevated serum ferritin levels on HSCT outcomes, including inferior overall survival rates. These findings are primarily attributable to the increase in non-relapse mortality in most studies^29, 39–41^. Infectious complications^42^, hepatic dysfunction^43^, or veno-occlusive disease^44^ are also reportedly related to elevated ferritin levels before HSCT. The exact mechanisms of the relationship between elevated serum-ferritin levels and HSCT outcomes have yet to be fully elucidated. Our study demonstrates high busulfan AUCs in patients with high ferritin levels, suggesting that high busulfan exposures could be a factor that increases toxicity after HSCT in patients with high ferritin levels.

One limitation of our study is the possibility that PK interactions between deferoxamine and busulfan cannot be excluded, as most of the patients with high ferritin levels received deferoxamine during the busulfan infusion. However, a gradual elevation or decline in the first day AUCs and clearance, based on ferritin levels, suggests that ferritin levels contribute to the increased AUCs.

Baseline urine levels of unsaturated medium-chain acylcarnitines were significantly lower in the high-AUC group than those of the low-AUC group in the present study. Acylcarnitines are fatty acid derivatives and are required for the transport of fatty acids into mitochondria, where the latter undergo β-oxidation and, hence, become essential intermediates in energy metabolism pathways. Unsaturated medium-chain fatty acids are primarily generated through mitochondrial β-oxidation. A small amount of these fatty acids escape from mitochondria and are converted to acylcarnitines to be secreted in the urine^45^. Previous studies have suggested that deregulation of mitochondrial fatty acid β-oxidation is associated with hepatic injury. In liver cancer patients, urinary acylcarnitines are reduced because of a decrease in mitochondrial fatty acid β-oxidation^45^. Serum metabolic profiling analyses in patients with chronic liver disease, including chronic hepatitis B and hepatic cirrhosis, showed accumulation of long-chain acylcarnitines, whereas the levels of free carnitine and short- and medium-chain acylcarnitines decreased depending on the severity of these non-malignant liver diseases^46^. Thus, low levels of baseline acylcarnitines related to high busulfan exposure may be indicative of hepatic mitochondrial dysfunction in patients with high busulfan exposure (Fig. 5).

Phenylacetylglutamine is formed via the conjugation of phenylacetate with l-glutamine by phenylacetyl CoA: l-glutamine N acetyltransferase, which is primarily found in the liver and kidneys. Phenylacetylglutamine is subsequently excreted in the urine and mediates the excretion of nitrogen^47^. Previous studies show that urinary phenylacetylglutamine is a marker for nitrogen waste scavenging^48^. Hyperammonaemia, a clinical condition associated with elevated ammonia levels, primarily causes liver cell damage. In this study, urinary phenylacetylglutamine levels were significantly lower in the high-AUC group than those of the low-AUC group. This finding suggests that pathways involved in ammonia metabolism could also affect busulfan exposure. Therefore, further studies are necessary to determine whether a potential correlation exists between ammonia levels and busulfan exposure (Fig. 5).

It is important to emphasise that all metabolites detected in this study were related to liver damage, indicating that liver function might affect busulfan exposure. The liver plays a central role in drug metabolism and elimination. Thus, altered hepatic drug metabolism attributable to liver dysfunction may result in high plasma drug concentrations and related toxicity. Currently, no well-established biomarkers of liver function related to drug elimination efficacy are available, warranting the search for biomarkers that are sensitive and specific enough to predict and/or explain liver function-related variability in busulfan exposure.

In conclusion, we found that busulfan metabolism is decreased in patients with reduced liver function using a pharmacometabolomic approach. Given that HSCT toxicity may be associated with high exposure to busulfan, a reduction in the busulfan dose should be considered for patients with reduced liver function and high ferritin levels to meet the target AUC. However, it should be noted that this study is an initial step in predicting busulfan exposure using a metabolomics approach, as it refers to data collected from a small number of patients. Although correlations between ferritin and busulfan clearance were evaluated in 130 paediatric patients, these results should be further validated before clinical implementation. Other metabolic markers should also be validated in a larger cohort of HSCT patients.

## Subjects and Methods

### Treatment and study

**Figure 6:**
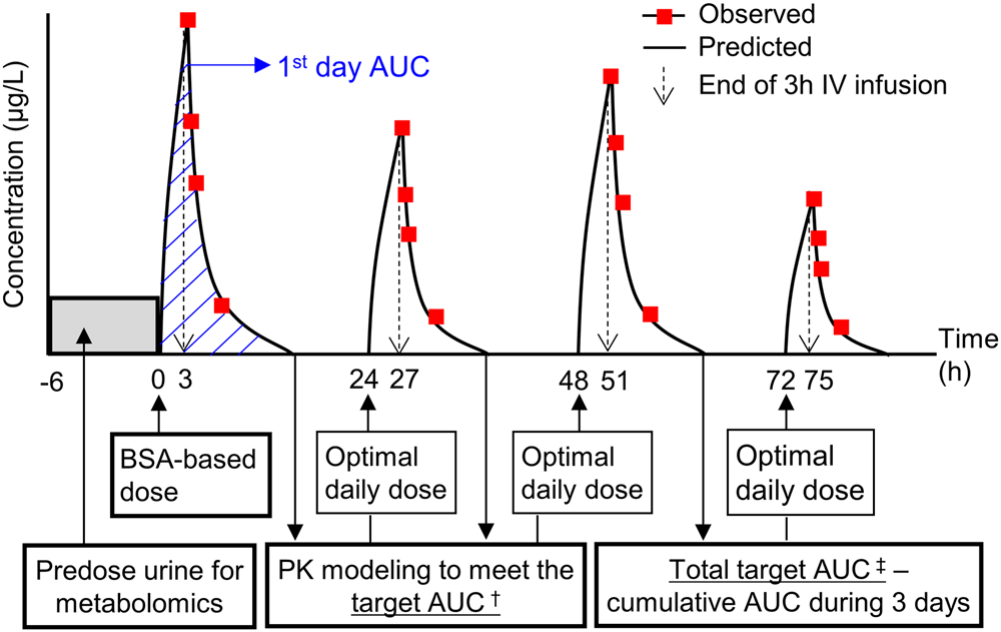
Dosing regimen scheme of busulfan in hematopoietic stem cell transplantation (HSCT) patients. Busulfan was administered once daily for 4 consecutive days and the area under the curve (AUC) was predicted from the pharmacokinetic (PK) modelling results. The solid line represents the predicted concentrations and open circles represent the observed concentrations at the sampling time (1, 2, and 4 h after the respective doses). According to the calculated AUC, the optimal dose was adjusted to meet the target AUC. ^†^18,000–19,000 μg∙h/L/day, ^‡^Sum of the AUC during 4 days (74,000 μg∙h/L).

Fifty-nine paediatric patients undergoing allogeneic HSCT using a busulfan-based conditioning regimen were enrolled. IV busulfan was administered over 3 h once daily for 4 consecutive days (Fig. 6). On the first day, initial busulfan dosing based on the Mosteller’s body surface area was used. Patients older than 1 year received 120 mg/m^2^, and patients younger than 1 year received 80 mg/m^2^. From the second day, we used a targeted dose of busulfan per the TDM results. To perform TDM, blood samples were taken after 3 h of busulfan infusions through a Hickman catheter line at 0, 1, 2, and 4 h, and the drug concentrations, AUC, and clearance were determined as previously described^49^. Briefly, AUC and clearance were calculated by one-compartmental methods using WinNolin 5.2.1 (Pharsight, Mountain View, CA). Target AUCs for the second and third day were 18,000–19,000 μg*h/L/day (4384–4628 μmol·min/L/day), and dose adjustments were done when AUCs were beyond that range. On the fourth day, the target AUC was calculated as 74,000 (cumulative AUC during 3 days) μg*h/L/day. Phenytoin (8 mg/kg/dose, q 12 h for 2 doses then q 24 h) was administered to prevent busulfan-induced seizures from 13.5 h before busulfan administration. Drugs that are known to influence busulfan PKs, such as itraconazole, fluconazole, metronidazole, and acetaminophen were avoided during busulfan administration. As shown in Fig. 6, 6-h interval urine samples for metabolic profiling were collected before the first busulfan administration. For children who had not completed toilet training, urine samples were collected using an attachable urine bag. Collected urine was stored in a container made of polyethylene and refrigerated for 1–2 h before freezing.

To validate the marker, the relationship between serum ferritin with the first day busulfan AUC or busulfan clearance was retrospectively analysed in 130 patients who underwent HSCT with a busulfan-based conditioning regimen. Serum ferritin levels were measured within 30 days of busulfan administration. This research was approved by the Institutional Review Board of Seoul National University Hospital (H-0911-050-301) and was conducted according to the principles expressed in the Declaration of Helsinki. Part of patients in this study were enrolled in clinical trials registered in the U.S. National Institute of Health clinical database (NCT01018446, registration date: November 20, 2009; NCT01274195, registration date January 10, 2011; NCT02047578, January 9, 2014; NCT02034630, January 9, 2014). Written informed consents were obtained from all patients.

### Metabolomics in urine

Urine samples were thawed on ice and prepared by centrifugation at 15,000 × *g* for 20 min at 4 °C to remove particles. Urine supernatant (50 μl) was diluted to 200 μL with distilled water. A sample aliquot (4 μL) was injected onto a reverse-phase 2.1 × 100 mm ACQUITY 1.8 μm HSS T3 column using a Waters ultra-performance liquid chromatography (UPLC) system. The gradient mobile phase comprised 0.1% formic acid water (A) and methanol containing 0.1% formic acid (B). Each sample was resolved for 20 min at a flow rate of 0.4 mL/min. The gradient consisted of 5% B for 1 min, 5–30% B over 1–8 min, 30–70% B over 8–13 min, and 95% B for 14 min (maintaining for 2 min). The samples were then equilibrated at 95% A for 3.5 min before the next injection. A Waters Xevo G2 time-of-flight mass spectrometer (TOF-MS) operated in both the positive ion and negative ion electrospray ionization (ESI^+^ and ESI^−^) mode was used. To obtain consistent differential variables, a pooled urine sample (QC) was prepared by mixing aliquots of individual samples. The prepared QC sample was acquired through a series of injections, and data were obtained by random injection.

The metabolomics data set was imported into EZinfo 2.0 software (Umetrics, Umea, Sweden) for multivariate analysis (pareto-scaled). PCA was performed to examine intrinsic variations within a group and to assess the clustering behaviour between groups. Clustering of QC samples in PCA was assessed to reveal the stability and reproducibility of the platform. Subsequently, PLS-DA was further used to maximize variations among groups and to determine the variables that contribute to this variation. Orthogonal PLS-DA analyses were performed, and the findings expressed in a loading S-plot representing two vectors of covariance (x-axis, P(loadings)) and correlation (y-axis, P(correlation)) were used for selection of biomarkers. VIP values for all the peaks were used as a coefficient for ion selection. VIP values >1.0 were considered potential biomarkers and subjected to identification.

### Quantification of biomarkers for global metabolomics

Three endogenous biomarkers of carnitine C9:1, carnitine C12:1-OH, and phenylacetylglutamine were semi-quantified using TargetLynx (Waters Corp.) In the absence of reference standards of unsaturated or hydroxy acylcarnitines, the related saturated acylcarnitines were used for the slope of the standard curve (i.e. carnitine C10:0 and carnitine C12:0 for carnitine C9:0 and carnitine C12:1-OH, respectively). Creatinine was also quantified for use as a normalisation standard for the actual concentration of each urinary biomarker. Urine supernatants (50 μL) were diluted with three international standard mixtures (150 μL) as follows: carnitine C10:0-*d*_3_ and carnitine C12:0-*d*_3_ (500 ng/mL), phenylacetylglutamine-*d*_5_, and creatinine-*d*_3_1 (1 μg/mL). The concentration of each biomarker in the urine was determined from the calibration curves using linear regression analysis. All determined correlation coefficients were >0.98 for each biomarker, and the resultant concentrations are expressed as μg/mg creatinine (normalised).

### Statistical analysis

Multivariate statistical analysis of metabolomics was performed using EZinfo 2.0 software (Umetrics, Umea, Sweden). PCA, PLS-DA, and orthogonal PLS-DA were used as classification methods to model discrimination by visualizing the score plot. Variables with VIP values >1.0 in the orthogonal PLS-DA models were sorted as potential biomarkers. To compare the concentrations of urinary biomarkers between the low-, medium-, and high-exposure groups, the Jonckheere-Terpstra trend test and Kruskal-Wallis test followed by Bonferroni-adjusted Mann-Whitney tests were applied using SPSS version 23.0 (IBM, USA, Armonk, NY).

Differences between means in continuous variables were calculated with the Student’s t-test or one-way ANOVA if the data followed normal distribution. When the data did not follow a normal distribution, non-parametric tests (Mann–Whitney U test or Kruskal-Wallis test) were used. Bivariate correlation analysis and the Pearson correlation coefficient were used to evaluate the association between continuous variables. Statistical significance was accepted when the *P*-value was less than 0.05.

## Electronic supplementary material

Supplementary Information
