# Supplementary material for: Pharmacometabolomics for predicting variable busulfan exposure in paediatric haematopoietic stem cell transplantation patients
Source: Sci Rep. 2017 May 10;7:1711. doi: 10.1038/s41598-017-01861-7 (PMC5431879; doi:10.1038/s41598-017-01861-7)

Supplementary Information

Pharmacometabolomics for predicting variable busulfan exposure in paediatric haematopoietic stem cell transplantation patients

Bora Kim[1,*], Ji Won Lee[2,*,#], Kyung Taek Hong[2], Kyung-Sang Yu[1], In-Jin Jang[1], Kyung Duk Park[2], Hee Young Shin[2], Hyo Seop Ahn[2], Joo-Youn Cho[1], and Hyoung Jin Kang[2]

[1]Department of Clinical Pharmacology and Therapeutics, Seoul National University College of Medicine and Hospital, Seoul, Korea; [2]Department of Pediatrics, Cancer Research Institute, Seoul National University College of Medicine and Hospital, Seoul, Korea.

*These authors equally contributed to this work.
#Current affiliation: Department of Pediatrics, Samsung Medical Center, Sungkyunkwan University School of Medicine

Corresponding Authors:
Hyoung Jin Kang, E-mail: kanghj@snu.ac.kr Phone: (82)2-2072-3304, Fax: (82)2-743-3455
Joo-Youn Cho, E-mail: joocho@snu.ac.kr Phone: (82)2-2072-3625, Fax: (82)2-743-3455

Supplementary Figures

Supplementary Figure S1. (**A**) Deferoxamine structure and its mass fragmentation. (**B**) MS/MS spectra of urinary deferoxamine (upper) and authentic deferoxamine (lower). The fragments and relative intensities of 16 urinary metabolites were compared with the fragmentation pattern of authentic deferoxamine.

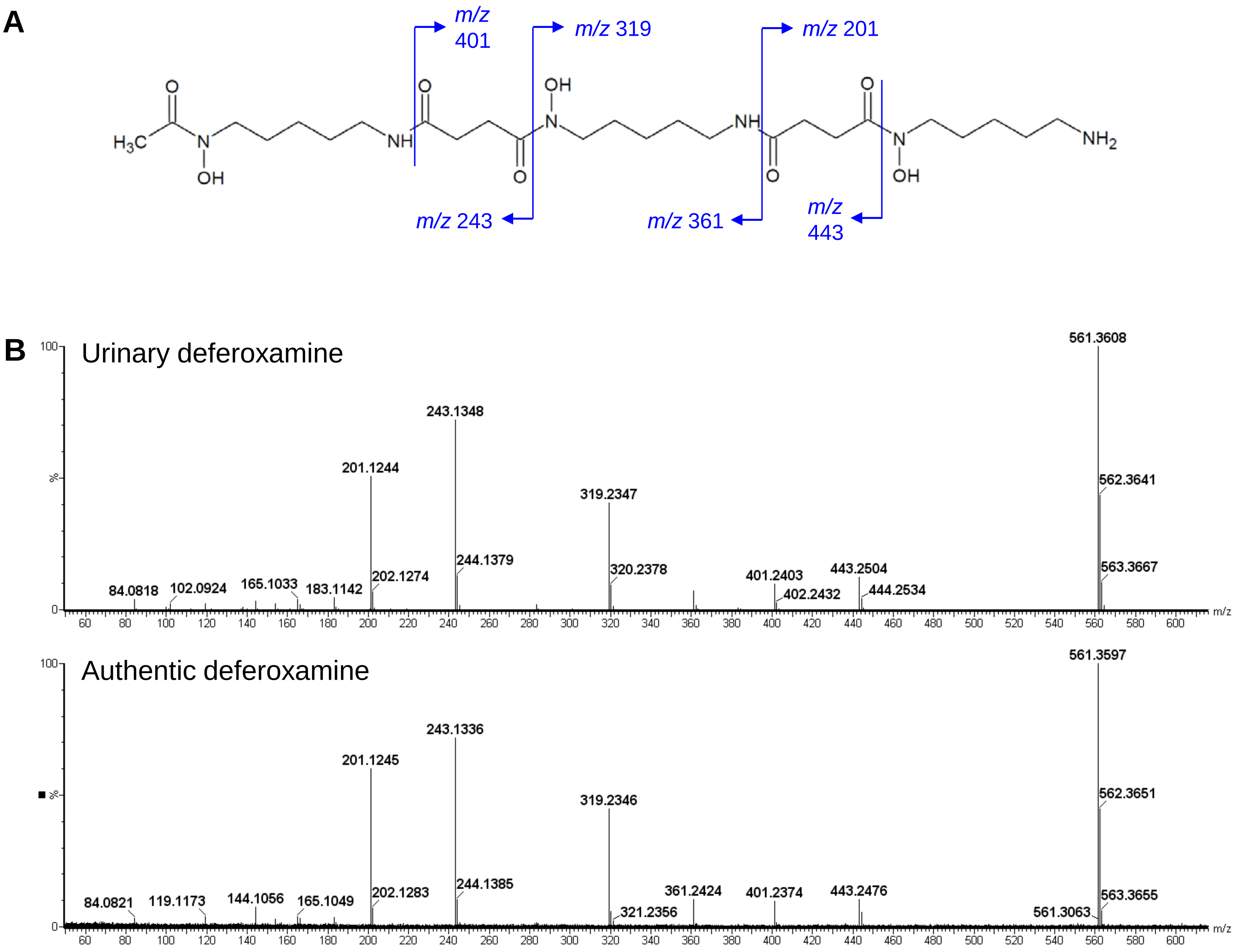
A
m/z 401
m/z 319
m/z 201
m/z 243
m/z 361
m/z 443
B
Urinary deferoxamine
561.3608
243.1348
201.1244
319.2347
562.3641
244.1379
320.2378
401.2403
443.2504
563.3667
202.1274
84.0818
102.0924
165.1033
183.1142
402.2432
444.2534
Authentic deferoxamine
561.3597
243.1336
201.1245
319.2346
562.3651
244.1385
202.1283
361.2424
401.2374
443.2476
563.3655
84.0821
119.1173
144.1056
165.1049
321.2356
561.3063
m/z
%

Supplementary Figure S2. The fragments of urinary C9:1 carnitine was compared with authentic C8:0 carnitine. MS/MS spectra and each structure of C9:1 carnitine and C8:0 carnitine.

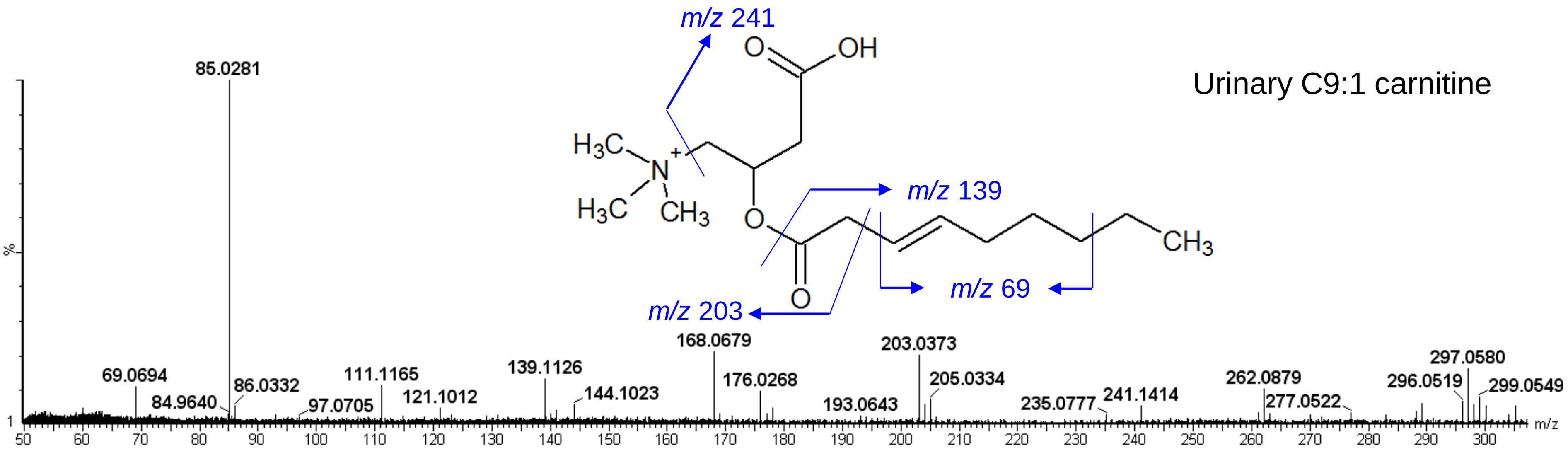

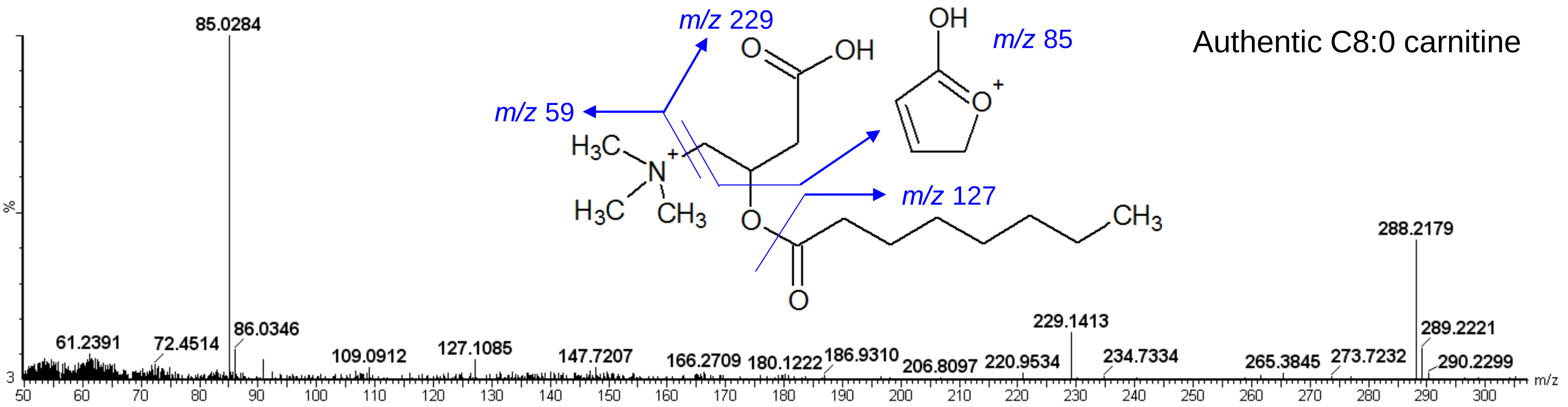

Supplementary Figure S3. The fragments of urinary C12:1-OH carnitine was compared with authentic C10:0 carnitine. MS/MS spectra and each structure of C12:1-OH carnitine and C10:0 carnitine.

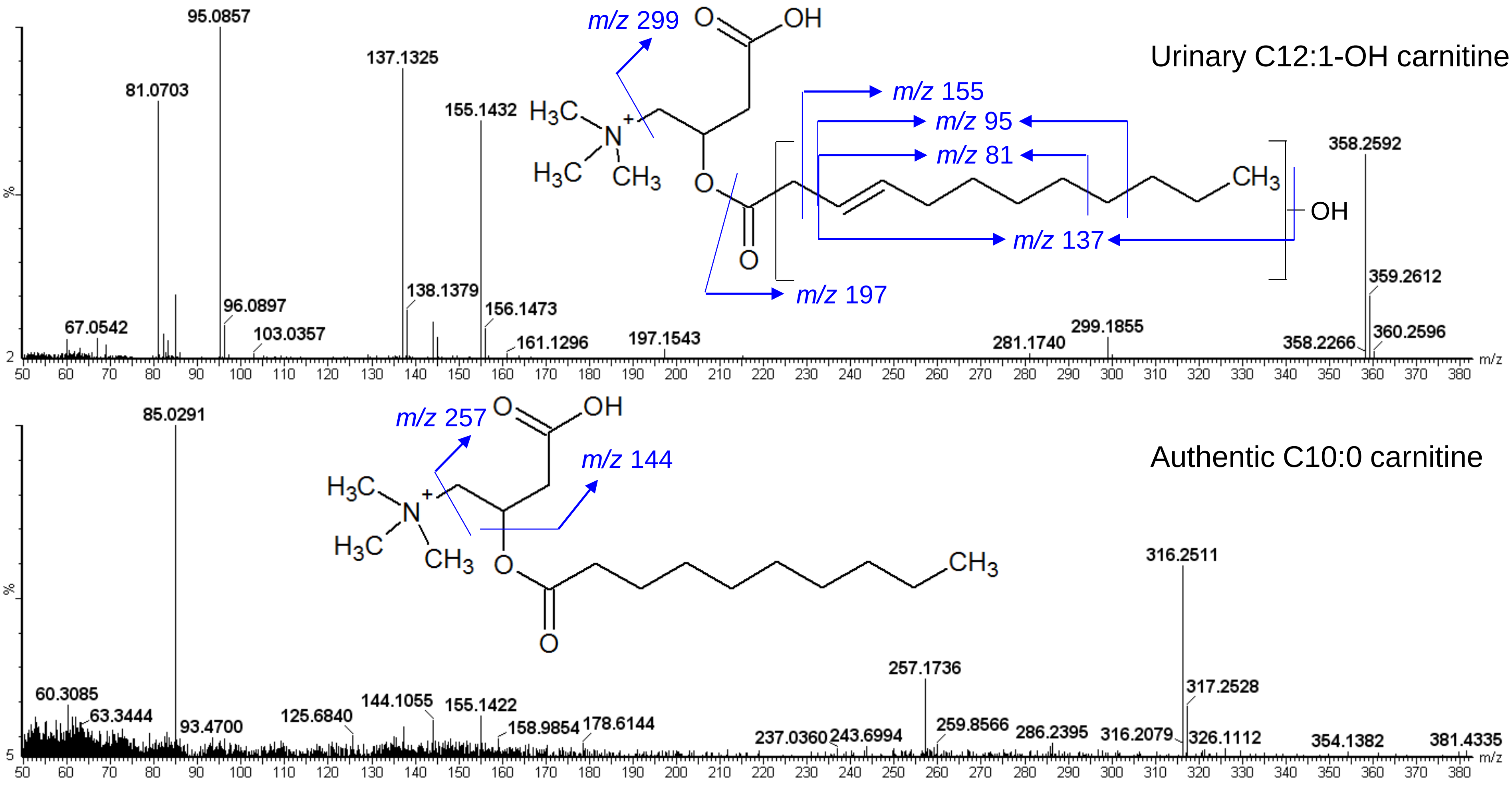

Supplementary Figure S4. Phenylacetylglutamine structure and MS/MS spectra of urinary PAGN, [M−H]− adduct, [2M+Na−2H]− adduct, and authentic PAGN.

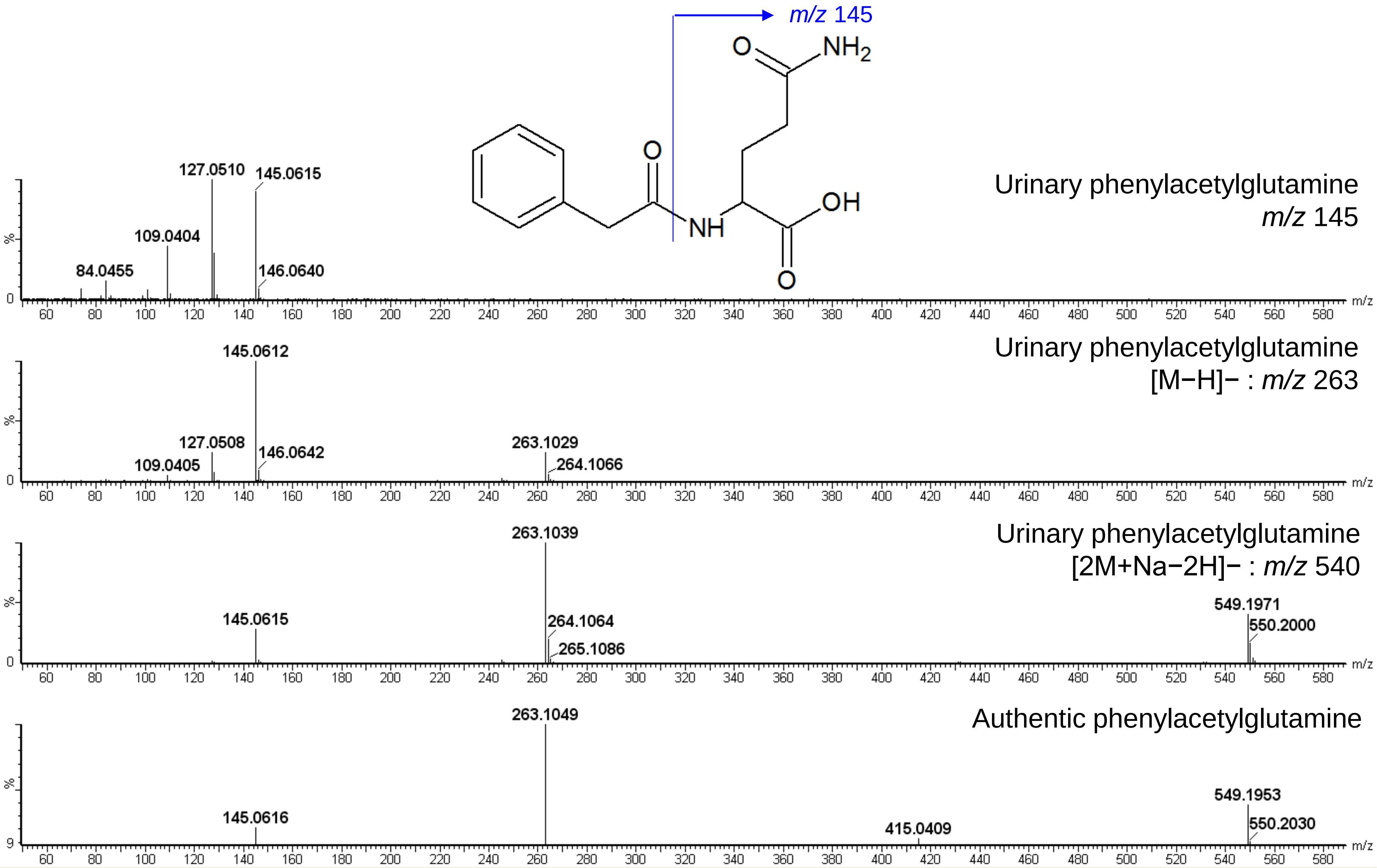

Supplement: Supplementary file 1 — Supplementary Information [file 41598_2017_1861_MOESM1_ESM.pdf]
